# Improving the Efficacy of Conventional Therapy by Adding Andrographolide Sulfonate in the Treatment of Severe Hand, Foot, and Mouth Disease: A Randomized Controlled Trial

**DOI:** 10.1155/2013/316250

**Published:** 2013-01-15

**Authors:** Xiuhui Li, Chi Zhang, Qingsheng Shi, Tong Yang, Qingxiong Zhu, Yimei Tian, Cheng Lu, Zhiying Zhang, Zhongsheng Jiang, Hongying Zhou, Xiaofeng Wen, Huasheng Yang, Xiaorong Ding, Lanchun Liang, Yan Liu, Yongyan Wang, Aiping Lu

**Affiliations:** ^1^You'an Hospital, Capital Medical University, Beijing, Fengtai 100069, China; ^2^China Academy of Chinese Medical Sciences, Beijing 100700, China; ^3^Handan Maternal and Child Health Hospital, Handan, Hebei 056001, China; ^4^The People's Hospital of Liuzhou, Liuzhou, Guangxi 545006, China; ^5^Children's Hospital of Jiangxi Province, Nanchang, Jiangxi 330006, China

## Abstract

*Background*. Herb-derived compound andrographolide sulfonate (called Xiyanping injection) recommended control measure for severe hand, foot, and mouth disease (HFMD) by the Ministry of Health (China) during the 2010 epidemic. However, there is a lack of good quality evidence directly comparing the efficacy of Andrographolide Sulfonate combination therapy with conventional therapy. *Methods*. 230 patients were randomly assigned to 7–10 days of Andrographolide Sulfonate 5–10 mg/Kg/day and conventional therapy, or conventional therapy alone. *Results*. The major complications occurred less often after Andrographolide Sulfonate (2.6% versus 12.1%; risk difference [RD], 0.94; 95% CI, 0.28–1.61; *P* = 0.006). Median fever clearance times were 96 hours (CI, 80 to 126) for conventional therapy recipients and 48 hours (CI, 36 to 54) for Andrographolide Sulfonate combination-treated patients (*χ* ^2^ = 16.57, *P* < 0.001). The two groups did not differ in terms of HFMD-cause mortality (*P* = 1.00) and duration of hospitalization (*P* = 0.70). There was one death in conventional therapy group. No important adverse event was found in Andrographolide Sulfonate combination therapy group. *Conclusions*. The addition of Andrographolide Sulfonate to conventional therapy reduced the occurrence of major complications, fever clearance time, and the healing time of typical skin or oral mucosa lesions in children with severe HFMD.

## 1. Introduction

Hand, foot, and mouth disease (HFMD) is a common infectious disease of children [1]. Some cases are mild and reversible, but severe cases show rapid progression to neurogenic pulmonary edema, pulmonary hemorrhage, and shock-induced sudden death with a higher frequency in young children [2]. In recent years, outbreaks of HFMD have increased, and more and more severe cases have appeared. In 2007, 0.9% of the Chinese cases were classified as severe [1]. After only three years, China experienced the largest outbreak on the record of HFMD with more than 1.7 million cases, 27,000 patients with severe neurological complications and 905 deaths [3]. And approximately 10%–30% of hospitalized cases during Enterovirus 71 associated HFMD epidemics in Asia have developed a spectrum of central nervous system complications, including aseptic meningitis and encephalitis [4–9]. 

Clinical management of HFMD is largely supportive in nature, and there are no specific antivirals [1, 9]. Therefore, the development of a more effective treatment for severe HFMD is a major goal. Chinese herbal medicines are widely used in treating HFMD in China [10]. In 2010, the Ministry of Health of China issued “Guideline for the diagnosis and treatment of hand foot and mouth disease,” and recommended series of Chinese herbal products for the treatment of HFMD including herbal injection [11]. Among them, the Herb-derived compound Andrographolide Sulfonate, approved for clinical use by State Food and Drug Administration (China) with promising efficacy in treating pediatric infectious diseases, is recommended for the treatment of HFMD. The Andrographolide was obtained from a popular Chinese herb Andrographis paniculata (Burm) Nees, which is used to low body temperature and for the detoxification in Chinese medicine. Andrographolide has many types of bioactivity, such as antivirus, anti-inflammatory, and antimicrobial [12]. It has an effectively antithermic and antibacterial action to the infection caused by multiple virus and bacteria [13, 14].

In the initial small size studies, Andrographolide Sulfonate was associated with reducing fever clearance time, rash subsidence time, and oral symptoms healing time [15–20]. A recent systematic review of Chinese herbal medicines for HFMD indicated that Chinese herbal medicines (especially herbal injections) and the combination of herbal medicine with western medications might improve symptoms of HMFD [21]. Both the literature review [22] and systematic review suggest that carefully designed prospective studies and high quality studies of sufficient sample size are needed.

Our goal was to test the hypothesis that the addition of Andrographolide Sulfonate to conventional therapy is more effective than conventional therapy alone for the treatment of severe HFMD. 

## 2. Methods

### 2.1. Setting and Participants

All eligible children were recruited between May 2010 and November 2010, from 3 centers in China located in Handan city (Hebei province), Nanchang city (Jiangxi province), and Liuzhou city (Guangxi province). The study was conducted in accordance with the Declaration of Helsinki, approved by local ethics committees and institutional review boards as appropriate and registered (clinical trials registration: NCT01554930).

All patients provided written informed consent prior to participating in the study. Eligible patients had to have had clinical diagnosis with severe HFMD patients according to guideline for the diagnosis and treatment of hand, foot, and mouth disease (2010) issued by the ministry of health of China [11]. In the absence of the World Health Organization standardized case definitions and guidelines for clinical management of severe cases, several countries developed their official guidelines [1, 11]. The guidelines issued by China emphasized the importance of diagnosis that is supported by clinical symptoms and laboratory diagnosis and defined severe HFMD patients presenting with obvious symptoms of nervous system involvement. Additional inclusion criteria were ages 1–13 years; the subjects' guardians are able to understand and sign the informed consent, no more than 24 hours after the occurrence of central nervous system symptoms, with any of the followings: lethargy and weakness, agitation or irritability, headache, vomiting, limb weakness or acute flaccid paralysis, myoclonic jerks, ataxia, nystagmus, and oculomotor palsies. We excluded patients who had been suffering from neurogenic pulmonary edema, heart, or respiratory failure. Other exclusion criteria were associated with other diseases such as chronic hepatitis, congenital heart disease, acute or chronic nephritis and blood diseases, a history of allergy in herbal medicine; a history of mild increases in bilirubin, with intravascular hemolysis (or uric bravery former positive), using hormonal therapy, attending other clinical studies on HFMD, and any condition that in the opinion of the investigator, may interfere with the evaluation of study objectives.

General practices receive one notification when a child is diagnosed with HFMD registered at the practice. Enterovirus isolation and identification were conducted in the Clinical Virology Laboratory of Beijing Ditan Hospital. Human EV71, Coxsackievirus A16, and Enterovirus general type Nucleotide Acid Detection Kit were selected for the identification (RT-PCR double Fluorescence Taqman probes, Daan Gene Company Limited, China). During the recruitment phase of the study, this notification was collated by assistants at each collaborating practice and reviewed by the trial investigators to exclude children who did not fulfill the eligibility criteria. 

### 2.2. Sample Size

Considering to only a few small size trials of Chinese herbal medicine for the HFMD performed in China, no formal power calculations were done for the pilot study, and severe HFMD is a relatively uncommon condition; the sample size (*n* = 230) was chosen to give a sufficient number to be able to draw reasonable inferences about enrollment, adherence, and loss to followup.

### 2.3. Randomization and Intervention

Before the baseline visit, the trial identification number, date of birth, and trial center were entered into a web-based randomization system. This system is provided by China Academy of Chinese Medical Sciences, which adopted the computer telephone integration technology to integrate computer, internet, and telecom. The random number list will be assigned by interactive voice response and interactive web response [23]. After eligibility had been determined and consent had been obtained, the researcher then immediately informed the assistants at each collaborating practice and provided children with further information about the allocated treatment. Andrographolide Sulfonate used in our study was light yellow to orange clear liquid, which is composed of total Andrographolide Sulfonate (manufactured by GMP certificated Jiangxi Qingfeng Pharmaceutical Inc.). The criteria for the quality of the injection used were in accordance with the Chinese pharmacopoeia (2005) [24]. Conventional therapy was administered in both groups of patients according to the guideline for the diagnosis and treatment of HFMD (China, 2010) [11], including decreasing intracranial hypertension, conscious sedation, reducing temperature, and applying glucocorticoids and immunoglobulin' intravenous. The detailed treatments included Mannitol 0.5–1.0 g/kg IV administered over 30–40 minutes, every 4 to 8 hours, glucocorticoid methylprednisolone (1-2 mg/kg/24 h), hydrocortisone (3–5 mg/kg/24 h); Dexamethasone (0.2–0.5 mg/kg/24 h), intravenous immunoglobulin (IVIG) 2 g/kg over 2 to 5 days (Recommended in patients with encephalitis plus acute flaccid paralysis; may be considered in patients with brainstem encephalitis), and others, like paracetamol, oxygen, and transfer to ICU if needed. In patients receiving combination therapy, Andrographolide Sulfonate was administered 5–10 mg/Kg/day for intravenous infusion (in 5% Dextrose). The treatments were given for 7–10 days. We followed patients in both groups every day in the treatment and in posttreatment period for 15 days. All 15-day followups were completed by November 2010.

### 2.4. Outcomes and Measurements

The primary outcome was one or more major complication (aseptic meningitis, brainstem encephalitis, encephalitis, purulent meningitis, encephalomyelitis, acute flaccid paralysis, autonomic nervous system dysregulation, pulmonary oedema/haemorrhage, respiratory failure, circulatory failure, cardiorespiratory failure, or any other serious adverse event). The secondary outcomes were the fever clearance time (fever clearance time was defined as the time to first drop in body temperature ≤37°C which remained ≤37.0°C for the subsequent 24 hours, after the first dose of an intervention has been given), HFMD-cause mortality, the healing time of typical skin or oral mucosa lesions (healing time was defined as the number of days in the total-contact cast until the skin completely closed), and the length of hospital stay and adverse event. Hospital care and discharge criteria were predefined using the guideline [11].

### 2.5. Statistical Analysis

All randomized subjects were assessed by comparing baseline characteristics of both study groups using unpaired *t*-tests for continuous variables and Chi-squared tests or Wilcoxon rank-sum test for categorical variables. The primary analysis was performed on the intent-to treat approach including all randomized patients with a baseline value and at least 1 treatment period measurement. The symptoms measured at baseline and 1–7 days were analyzed using Wilcoxon rank-sum test at endpoint. Missing data for secondary endpoints were imputed using the last observation carried forward method. Unless otherwise stated, results are reported as means (SD). Assumptions of normality and homoscedasticity were assessed. All statistical tests were 2 tailed, and *P* values less than 0.05 were considered statistically significant. The safety evaluation included all randomized patients. The number and percentage of patients reporting clinical adverse experiences were summarized by treatment group. All statistical procedures were performed with SAS PROC LCA (version 9.2, SAS Institute, Inc., Cary, NC, USA).

### 2.6. Role of the Funding Source

State Administration of Traditional Chinese Medicine of China provided peer reviewed funding for this study. The sponsors of this study had no role in study design, data collection, data analysis, data interpretation, or writing of the report. The corresponding author had full access to all the data in the study and had final responsibility for the decision to submit for publication.

## 3. Results

### 3.1. Participant Characteristics

**Figure 1:**
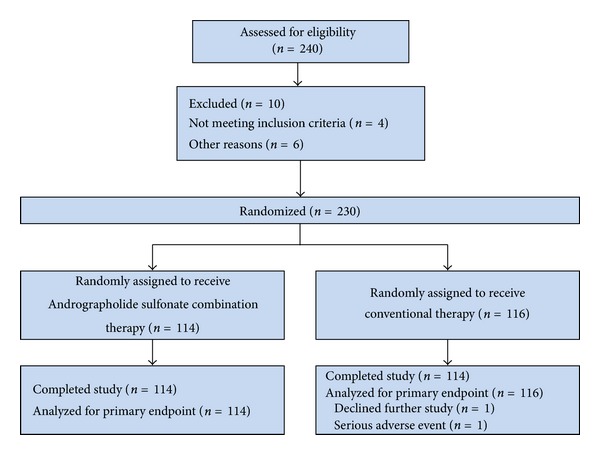
Flow of participants with severe HFMD through a randomized trial comparing the efficacy of Andrographolide Sulfonate combination therapy with conventional therapy alone.

A total of 116 and 114 patients were randomly assigned to conventional therapy alone and Andrographolide Sulfonate combination groups, respectively. All patients received the allocated treatment for the efficacy analysis and received complete courses until the designated endpoints were reached (Figure 1).

Baseline demographics and clinical characteristics were well matched between groups (Table 1). The Enterovirus 71 isolation diagnosis was proven in 137 (59.6%) of 230 patients, and CA16 was found in 7 (3.0%) patients, and 53 cases (23.0%) remained without any known information about the virus isolation. 

### 3.2. Clinical Outcomes

There were statistical differences in outcomes including major complications, the fever clearance time, and the healing time of typical skin or oral mucosa lesions (Table 2).

Of 230 patients, 17 complications (7.4%) were noted. The major complications occurred less often after Andrographolide Sulfonate (2.6% versus 12.1%; risk difference [RD], 9.4; 95% CI, 2.8–16.1; *P* = 0.006). New-onset circulatory failure, purulent meningitis, brainstem encephalitis, encephalomyelitis, neurogenic pulmonary edema and pulmonary haemorrhage did not occur after Andrographolide Sulfonate combination therapy (0% versus 0.9%; 0.9; 95% CI, −0.8–2.5; *P* = 1.00). Fewer patients after Andrographolide Sulfonate developed respiratory failure (1.8% versus 4.3%; 2.6; 95% CI, −1.9–7.0; *P* = 0.45) and encephalitis (0.9% versus 1.7%; 0.9; 95% CI, −2.1–3.8; *P* = 1.00).

**Figure 2:**
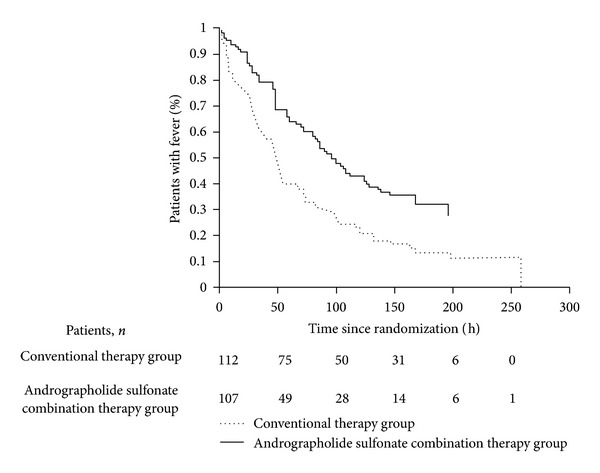
Kaplan-Meier curves of time from the start of treatment to the recording of a temperature ≤37.0°C which remained ≤37.0°C for the subsequent 24 hours for conventional therapy (dotted line) and Andrographolide Sulfonate combination therapy (solid line) for the treatment of severe HFMD. Log-rank test *χ* ^2^ = 16.57; *P* < 0.001.

The mean (SD) body temperature of both randomization groups from the initial screen to the 7 day followup are shown in Supplementary Figure 1 available online on http://dx.doi.org/10.1155/2013/316250. Both groups had similar mean scores at the initial body temperature screen and at the visit eligibility assessment, but the improvement were greater in the Andrographolide Sulfonate combination therapy group at the followup. The median time to fever clearance time was 96 hours (CI, 80 to 126) for conventional therapy recipients and 48 hours (CI, 36 to 54) for Andrographolide Sulfonate combination-treated patients (*χ* ^2^ = 16.57, *P* < 0.001) (Figure 2). The fever clearance time was reduced 27.8 hours (CI, 11.2 to 44.4) with Andrographolide Sulfonate combination therapy compared with only conventional therapy (*P* < 0.001). 

HFMD mortality rate of Andrographolide Sulfonate combination therapy group (0%) was lower than of conventional therapy group (0.86%), but the difference did not reach to significant *P* value (*P* = 1.00). 

No statistical difference (*P* = 0.70) in the duration of hospital stay was found between the two groups (7.6 ± 2.0 days versus 7.7 ± 1.7 days).

The healing time of typical skin or oral mucosa lesions was 4.3 ± 1.5 days in the Andrographolide Sulfonate plus conventional therapy group and 5.2 ± 1.6 days in conventional therapy group. The difference was statistically significant (*P* < 0.001).

### 3.3. Safety

Andrographolide Sulfonate was well tolerated. Among all patients who received conventional therapy, there was one death, which was induced by the development of complications during treatment. This 47-month-old boy presented to the hospital with a 2-day history of fever. At admission, he had a temperature of 37.5°C, a heart rate of 135 beats/min, a respiratory rate of 37 breaths/min, and a blood pressure of 98/50 mm Hg. He was drowsy and had a papular rash. The patient was diagnosed with HFMD and brainstem encephalitis. Despite therapy with diuretics, inotropic agents, and intravenous immunoglobulin, the patient developed progressive respiratory failure, resulting in death 1 day after admission. Beyond that, there was no adverse event observed in both groups. 

## 4. Discussion

This randomized, controlled trial demonstrates that Andrographolide Sulfonate combined with conventional therapy significantly reduces incidence of major complications, accelerates fever clearance time and the healing time for shin or oral mucosal lesions. No deaths and severe adverse events were seen in Andrographolide Sulfonate combination therapy group during a 15 day followup duration. 

We have compared the outcomes from our trial with those of other comparable studies, identified from a recent review [21], WHO HFMD guidelines [1], and a search of Medline using these terms: HFMD, trial. Previous meta-analysis showed that compared to ribavirin alone, Andrographolide Sulfonate plus ribavirin had better effect in reducing rash subsidence time and the oral ulcers healing time. Similarly, our findings are consistent with the results of previous trials. Although previous randomized controlled trials have attempted to investigate the effects of Andrographolide Sulfonate, most of the past trials used composite outcome measures which categorized the effect of the treatment into four grades. The classification of “cure,” “markedly effective,” “effective,” or “ineffective” is highly subjective. Thus, our prospective study provides strong evidence for Andrographolide Sulfonate combination treatment.

Andrographolide, chemically designated as 2(3H)-furanone1, 3-(2-(decahydro-6-hydroxy-5-(hydroxymethyl)-5,8a-dimethyl-2-methylene-1-napthalenyl)ethylidene) dihydro-4-hydroxy-, was one of main active constituents of Andrographis paniculate (Burm) Nees, a famous Chinese herb characterized by removing heat and toxic marerials and relieving inflammation (12); it is possible that this mechanism is responsible for the clinical improvement that is observed. The preparations of andrographolide that we used are those that have been standard for decades, but their efficacy and safety for HFMD hve not been clearly investigated. In our study, no adverse event was reported in the Andrographolide Sulfonate combination group. This agrees with other studies [25] and echoes findings from State Food and Drug Administration (China) on no adverse event report in May 2008. However, a recent study suggests about 26.1 percent of the adverse drug reactions in China for HFMD are related to herbal injections, which rank second only to antibiotic shot [26]. Furthermore, State Food and Drug Administration released a no. 48 “Adverse Drug Reaction Information Bulletin,” the National Adverse Drug Reaction Monitoring Center prompted Andrographolide Sulfonate caused allergic reaction [27]. Despite, we did not report unwanted events, this finding was at odds with observations on HFMD patients with skin rash, possibly because doctors were more likely to report rash due to HFMD. Similarly, vomiting, diarrhea, nausea, and headache were common in both groups, whereas they were some of the common symptoms of HFMD, the majority of these events were mild to moderate in severity; we did not record these events as adverse events suspected of being associated with the medications.

There were a number of limitations related to this study. This study lacked a double-blind design, and 230 patients were all Chinese from 3 provinces in China, which may limit generalizability. Another limitation of this study was that like all published trials did not report long-term followup after the treatment, we only collected outcome measures for 15 days after enrollment in all patients. On the other hand, due to the lack of long-term followup, it's hard to conclude the safety of Andrographolide Sulfonate on HFMD. And it could also be argued that Enterovirus isolated in our study was insufficient, and 53 cases remain unproven because they refused to provide the blood sample at three time points, 1, 3, 5, and 7 days, though they were definitely diagnosed with severe HFMD according to the guideline [11]. Furthermore, our study was not adequately powered to detect differences between patients in the conventional therapy. Variability of the success of the therapy may have influenced the amount of discomfort experienced, and subsequently the central nervous system profiles exhibited by the patients. 

## 5. Conclusions

In summary, the findings are consistent with the recommendation published by the Ministry of Health of China that Andrographolide Sulfonate can be used in the treatment protocol of HFMD. Based on the present study, we concluded that Andrographolide Sulfonate is a good choice as add-on therapy to conventional therapy for the treatment of severe HFMD. 

## Supplementary Material

Body temperature changes among patients who received Andrographolide Sulfonate combination therapy (solid line) versus patients who received conventional therapy (dotted line) from baseline to 7 days after treatment.

## Figures and Tables

**Table 1:** Characteristics for participants with severe HFMD involved in a randomized trial comparing the efficacy of Andrographolide Sulfonate combination therapy with conventional therapy alone.

Characteristic	Conventional therapy group (*n* = 116)	Andrographolide Sulfonate combination therapy group (*n* = 114)
Male, *n* (%)	76 (66.1)	77 (67.5)
Mean age (SD), m	25.7 (12.6)	26.0 (14.2)
Temperature, *n* (%)		
≤37.0°C	35 (30.17)	27 (23.68)
37.1–37.5°C	28 (24.14)	31 (27.19)
37.6–38.0°C	24 (20.69)	22 (19.30)
38.1–38.5°C	16 (13.79)	20 (17.54)
38.6–39.0°C	8 (6.90)	10 (8.77)
>39.0°C	5 (4.31)	4 (3.51)
Typical symptom, n (%)		
Skin or oral mucosa lesions*	112 (96.6)	105 (92.1)
Enterovirus isolated, n (%)		
EV 71	64 (55.2)	73 (64.0)
Cox A16	5 (4.3)	2 (1.8)
Others	3 (2.6)	3 (2.6)
Unproven	26 (22.4)	27 (23.7)
Height (SD), cm	85.53 (11.9)	82.87 (11.7)
Weight (SD), kg	13.44 (6.6)	13.77 (7.2)

*The skin or oral mucosa lesions varied among the case patients and were papulovesicular or maculopapular without vesicles.

**Table 2:** Outcomes for participants with severe HFMD involved in a randomized trial comparing the efficacy of Andrographolide Sulfonate combination therapy with conventional therapy alone.

Variable	Conventional therapy group (*n* = 116)	Andrographolide sulfonate combination therapy group (*n* = 114)	Treatment difference (95% CI)	*P* value
Primary Major complications, *n* (%)	14 (12.1)	3 (2.6)	9.4 (2.8 to 16.1)	0.006
Respiratory failure, *n* (%)	5 (4.3)	2 (1.8)	2.6 (−1.9 to 7.0)	0.45
Circulatory failure, *n* (%)	1 (0.9)	—	0.9 (−0.8 to 2.5)	1.00
Purulent meningitis, *n* (%)	1 (0.9)	—	0.9 (−0.8 to 2.5)	1.00
Brainstem encephalitis, *n* (%)	1 (0.9)	—	0.9 (−0.8 to 2.5)	1.00
Encephalomyelitis, *n* (%)	1 (0.9)	—	0.9 (−0.8 to 2.5)	1.00
Encephalitis, *n* (%)	2 (1.7)	1 (0.9)	0.9 (−2.1 to 3.8)	1.00
Neurogenic pulmonary edema, *n* (%)	2 (1.7)	—	1.7 (−0.6 to 4.1)	0.50
Pulmonary haemorrhage, *n* (%)	1 (0.9)	—	0.9 (−0.8 to 2.5)	1.00
Secondary				
Fever clearance time (SD), h*	96.9 (63.2)	69.1 (62.4)	27.8 (11.2, 44.4)	0.001
HFMD-cause deaths, *n* (%)	1 (0.86)	—	0.9 (−0.8 to 2.5)	1.00
Duration of hospitalization (SD), *d* ^†^	7.6 (2.0)	7.7 (1.7)	−0.1 (−0.5 to 0.4)	0.70
The healing time of typical skin or oral mucosa lesions (SD), *d* ^‡^	5.2 (1.6)	4.3 (1.5)	1.0 (0.6 to 1.4)	<.001
Adverse event, *n* (%)	1 (0.86)	—	0.9 (−0.8 to 2.5)	1.00

*Values are means with standard deviations.

^†^Surviving patients only.

^‡^defined as the number of days in the total-contact cast until the skin or oral mucosa completely closed.
